# Seconds-resolved pharmacokinetic measurements of the chemotherapeutic irinotecan *in situ* in the living body†
†Electronic supplementary information (ESI) available. See DOI: 10.1039/c9sc01495k


**DOI:** 10.1039/c9sc01495k

**Published:** 2019-07-22

**Authors:** Andrea Idili, Netzahualcóyotl Arroyo-Currás, Kyle L. Ploense, Andrew T. Csordas, Masayasu Kuwahara, Tod E. Kippin, Kevin W. Plaxco

**Affiliations:** a Department of Chemistry and Biochemistry , University of California, Santa Barbara , Santa Barbara , CA 93106 , USA . Email: kwp@ucsb.edu; b Center for Bioengineering , University of California, Santa Barbara , Santa Barbara , CA 93106 , USA; c Department of Pharmacology and Molecular Sciences , Johns Hopkins School of Medicine , Baltimore , Maryland 21205 , USA; d Department of Psychological and Brain Sciences , University of California, Santa Barbara , Santa Barbara , CA 93106 , USA; e Graduate School of Integrated Basic Sciences , Nihon University , 3-25-40 Sakurajosui, Setagaya-ku , Tokyo 156-8550 , Japan; f Department of Molecular Cellular and Developmental Biology , University of California, Santa Barbara , Santa Barbara , CA 93106 , USA; g Department of Neuroscience Research Institute , University of California, Santa Barbara , Santa Barbara , CA 93106 , USA

## Abstract

The ability to measure drugs in the body rapidly and in real time would advance both our understanding of pharmacokinetics and our ability to optimally dose and deliver pharmacological therapies. To this end, we are developing electrochemical aptamer-based (E-AB) sensors, a seconds-resolved platform technology that, as critical for performing measurements *in vivo*, is reagentless, reversible, and selective enough to work when placed directly in bodily fluids. Here we describe the development of an E-AB sensor against irinotecan, a member of the camptothecin family of cancer chemotherapeutics, and its adaptation to *in vivo* sensing. To achieve this we first re-engineered (*via* truncation) a previously reported DNA aptamer against the camptothecins to support high-gain E-AB signaling. We then co-deposited the modified aptamer with an unstructured, redox-reporter-modified DNA sequence whose output was independent of target concentration, rendering the sensor's signal gain a sufficiently strong function of square-wave frequency to support kinetic-differential-measurement drift correction. The resultant, 200 μm-diameter, 3 mm-long sensor achieves 20 s-resolved, multi-hour measurements of plasma irinotecan when emplaced in the jugular veins of live rats, thus providing an unprecedentedly high-precision view into the pharmacokinetics of this class of chemotherapeutics.

## Abstract 2

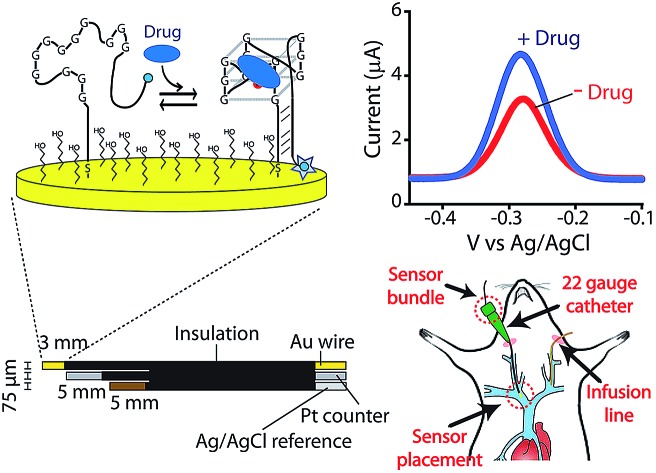
The ability to measure drugs in the body rapidly and in real time would advance both our understanding of pharmacokinetics and our ability to optimally dose and deliver pharmacological therapies.

## Introduction

The goal of personalized medicine is to precisely tailor treatment to the individual.^1^,^2^ To this end, an ability to measure drugs in the living body with seconds resolution would allow clinicians to define drug dosing based on high-precision, patient-specific pharmacokinetic measurements rather than on indirect predictors of drug metabolism such as age, body mass, or pharmacogenetics.^3^,^4^ Ultimately, the ability to measure drugs in the body in real-time would enable closed-loop feedback-controlled delivery,^5^ vastly improving dosing precision by actively responding to minute-to-minute fluctuations in a patient's metabolism.^4^,^6^ The development of such technology, however, faces significant hurdles.^7^,^8^ First, an *in vivo* sensor must be small enough to be placed in the body without causing undue damage. Second, it cannot require the addition of exogenous reagents or the use of batch processing, such as washing or separations. Third, it must make measurements at a frequency that is rapid relative to the drug's pharmacokinetics. Finally, it must be selective and stable enough to work for prolonged periods in the complex, fluctuating environments found *in vivo*. To this end we are developing electrochemical aptamer-based (E-AB) sensors, a technology that, by achieving these goals, supports the high frequency, real-time measurement of specific molecules directly in the living body.^9^

**Fig. 1:**
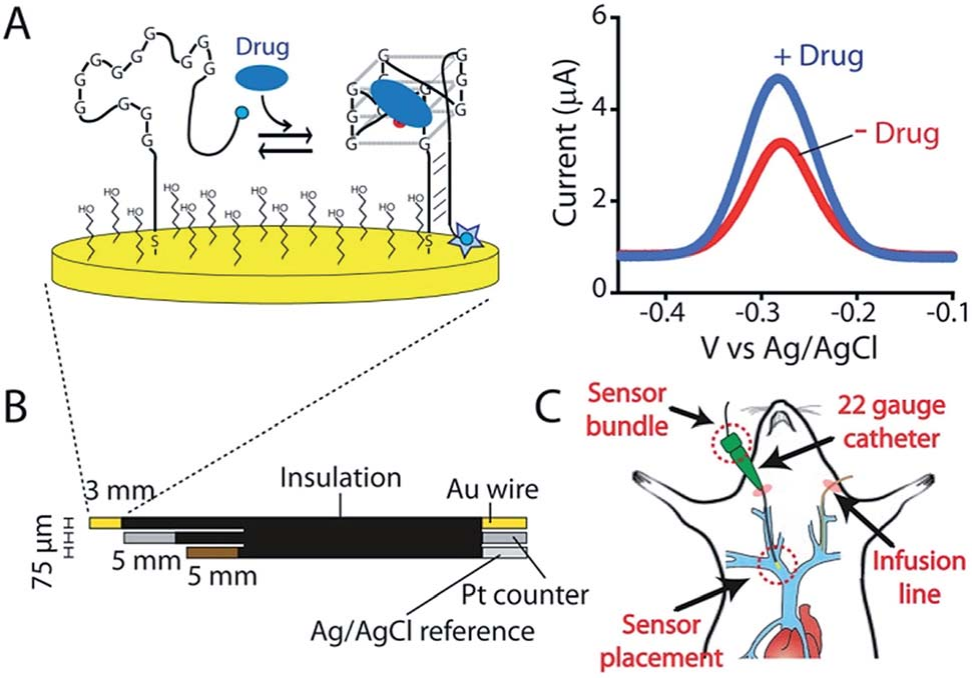
Indwelling E-AB sensors supporting the high-frequency measurements of plasma irinotecan levels *in situ* in the living body. (A) Electrochemical aptamer-based (E-AB) sensors consist of a redox-reporter-modified aptamer covalently attached to a gold electrode *via* an alkane-thiol self-assembled monolayer. In the absence of its specific target, the aptamer is partially or entirely unfolded (left). Target binding induces a conformational change, altering the efficiency with which the redox reporter (here a methylene blue molecule) approaches the electrode and thus altering electron transfer and (right) the signal observed upon voltammetric (here square-wave) interrogation. (B) In the completed sensor a 75 μm gold-wire working electrode is bundled with same-diameter platinum counter and a silver/silver-chloride reference electrodes, creating a device small enough and flexible enough to (C) be emplaced *via* a 22-gauge guide catheter in one of the external jugular veins of a live rat.

E-AB sensors employ an electrode-bound, redox-reporter-modified aptamer as their recognition element (Fig. 1A). Binding of the target molecule to this aptamer induces a conformational change that produces an easily measured electrochemical output (here we employ square wave voltammetry) without needing reagent additions or wash steps. Because E-AB signaling is generated by a binding-induced conformational change and not, as is the case for most other reagentless biosensor architectures, by the adsorption of target to the sensor surface,^7^ E-AB sensors are largely insensitive to non-specific adsorption and support multi-hour measurements in biological fluids not only *in vitro*^10^ but also *in vivo*.^9^ Finally, because their signaling arises due to target binding alone, and not, as is the case, for example, of the continuous glucose monitor,^11^ from the chemical reactivity of the target, E-AB sensors are a platform technology generalizable to a wide variety of analytes, including two of which, the aminoglycosides and doxorubicin, have been measured *in vivo*.^9^ Building on this foundation we describe here the fabrication and characterization of an E-AB sensor adapted to measurements *in situ* in the body, one directed against the camptothecin family of anticancer drugs, an important class of chemotherapeutic agents used in the treatment of a range of human cancers.^12^,^13^

## Results and discussion

**Fig. 2:**
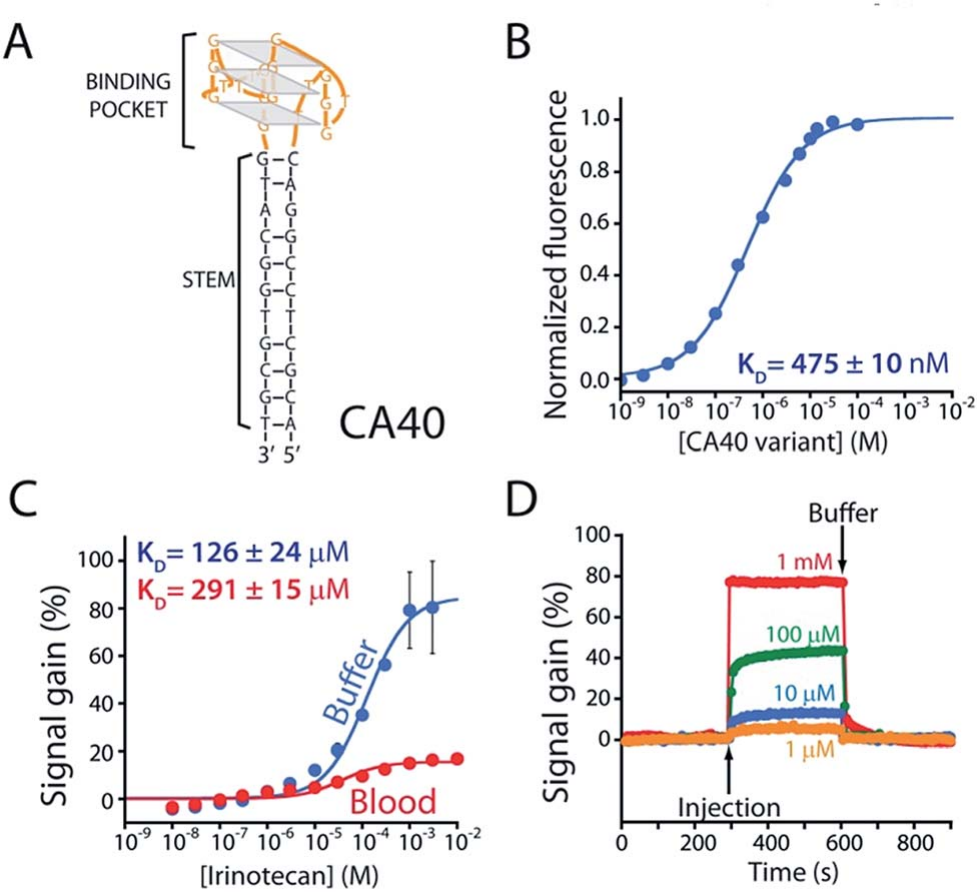
(A) The parent aptamer (CA40) is predicted to fold into a G-quadruplex, which is thought to be the target-binding site,^14^,^15^ flanked by a 12-base-pair stem. (B) Exploiting the intrinsic fluorescence of irinotecan (Fig. S1†) we find that, when free in solution, the aptamer exhibits a dissociation constant of 475 nM. (C) When redox-reporter-modified and anchored to the sensor's interrogating electrode, however, its affinity and signal gain are reduced significantly, particularly when deployed in undiluted whole blood. (D) The E-AB sensor nevertheless rapidly responds to when challenged (here in buffer) with irinotecan. Binding curves in panel C employed a square-wave frequency of 120 Hz. The kinetic experiments in panel D employed a square-wave frequency of 500 Hz and a repetition rate of 0.2 Hz.

As the recognition element in our sensor we employ a DNA aptamer that binds to the camptothecins.^14^,^15^ Specifically, a 40-base version of this aptamer, termed CA40, which folds into a target-recognizing G-quadruplex flanked by a 12-base-pair stem (Fig. 2A), binds the camptothecin, irinotecan, with a dissociation constant of 475 ± 10 nM when the unmodified aptamer is free in solution (Fig. 2B and S1†). To adapt this into an E-AB sensor modified its 3′ end with a methylene blue redox reporter and deposited it onto a gold electrode *via* a six-carbon thiol at its 5′ end (Fig. 1A). Electrochemically interrogating the resulting sensor in buffer we observe the expected Langmuir isotherm binding with an estimated *K*_D_ of 126 ± 24 μM (error bars here and, unless otherwise noted, reflect the standard deviation derived using at least three independently fabricated sensors), signal gain (the relative change in signal upon the addition of saturating target) of 84 ± 4% (Fig. 2C, blue curve) and association and dissociation kinetics too rapid to measure (time constants < 5 s at clinically relevant concentrations; Fig. 2D). We presume that the poorer affinity the aptamer exhibits in the context of the sensor arises due to interactions with the electrode surface, as this is known to destabilize the folding (thus hindering binding) surface-attached oligonucleotide.^16^ Despite this, when challenged in buffer the sensor supports the detection of irinotecan over the clinically relevant 1 to 15 μM range.^17^,^18^ When challenged in whole blood, however, the (apparent) affinity of the surface-bound aptamer is poorer still (*K*_D_ = 291 ± 15 μM), presumably because the concentration of the free drug is reduced due to protein binding.^17^ Worse, under these conditions the gain falls to 15 ± 1%, pushing its useful dynamic range out of the clinically relevant concentration window (Fig. 2C, red curve).

**Fig. 3:**
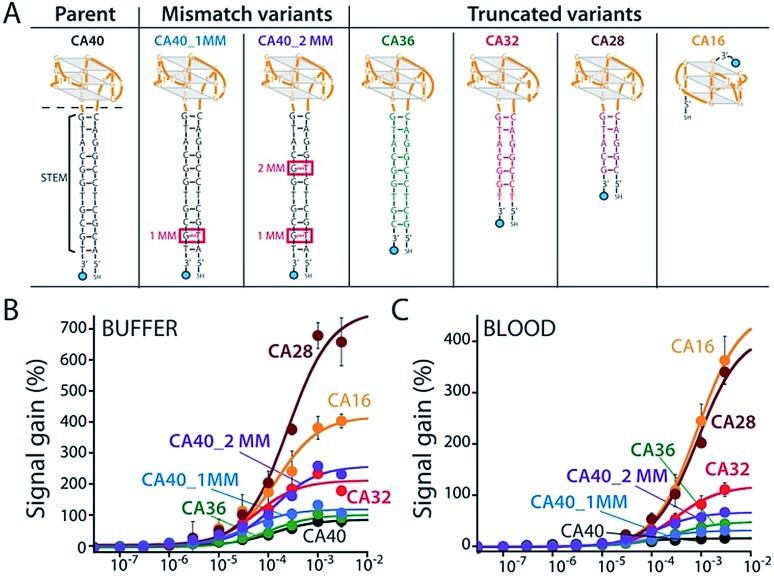
We reengineered the parent aptamer to produce higher-gain E-AB signaling. (A) We did so by destabilizing the aptamer's stem-loop (thus increasing the population of unfolded molecules poised to respond to target) *via* either introduction of one (CA40_1MM) or two (CA40_2MM) mismatches or *via* truncation (CA36, CA32, CA28, CA16) of the stem. (B) When challenged in a simple buffered solution all of the re-engineered variants exhibited higher gain than that of the parent aptamer (see ESI Table 1†), with the most destabilized (CA40_2MM, CA32, CA28, CA16) producing the greatest signal gain. (C) When tested in whole blood their gain and affinity are reduced, but the best performing nevertheless still support high-gain E-AB sensing.

To improve sensor performance in whole blood we reengineered the camptothecin-binding aptamer to better populate its “unfolded” state in the absence of target, thus increasing the sensor's gain.^19^,^20^ To do so we destabilized the aptamer's double-stranded stem (Fig. 3A) *via* either truncation (CA36, CA32, CA28, CA16) or the introduction of one (CA40_2MM) or two (CA40_2MM) G-T mismatches. As estimated using the nucleic acid folding predictor NUPACK^21^ these strategies should decrease the stability of folded aptamer from the –31.8 kJ mol^–1^ of the C40 parent to as low as –0.3 kJ mol^–1^ for CA28 (Fig. S2†). Characterizing sensors fabricated using these variants (Fig. 3A) we obtain dissociation constants ranging from 38 ± 11 μM to 254 ± 48 μM (Table 1 ESI†) and signal gain of up to 755% (Fig. 3B and Table S1†) when challenged in working buffer. Testing in whole blood (Fig. 3C) once again reduces both gain and apparent affinities (Table 1 ESI†). Even under these more challenging conditions, however, sensors employing the CA32, CA28, and CA16 variants support high-gain E-AB sensing.

Having achieved good *in vitro* performance with a sensor employing the CA32 variant we next set out to adapt this to use *in situ* in the veins of live rats. Under such conditions E-AB sensors often exhibit significant baseline drift.^9^,^22^ We have previously corrected this using “Kinetic Differential Measurements” (KDM) an approach that exploits the generally strong square-wave frequency dependence of E-AB signal gain.^9^,^22^ Specifically, the signal gain of the E-AB sensors we have previously described is so great that they exhibit a “signal-on” (target binding increases the signaling current) response at some square-wave frequencies no observable gain or even “signal-off” behavior at others.^9^,^22^ Conveniently, the signals obtained under these different regimes drift in concert such that taking their difference *via* KDM removes the drift seen *in vivo*.^9^ And since the two signaling currents respond in opposition in the presence of their target, taking their difference also improves signal gain. Uniquely in our experience, however, the gain of the camptothecin-detecting E-AB sensors (CA32, CA28, and CA16) is only a weak function of square-wave frequency (Fig. S3†), necessitating the development of a new approach to performing KDM.

**Fig. 4:**
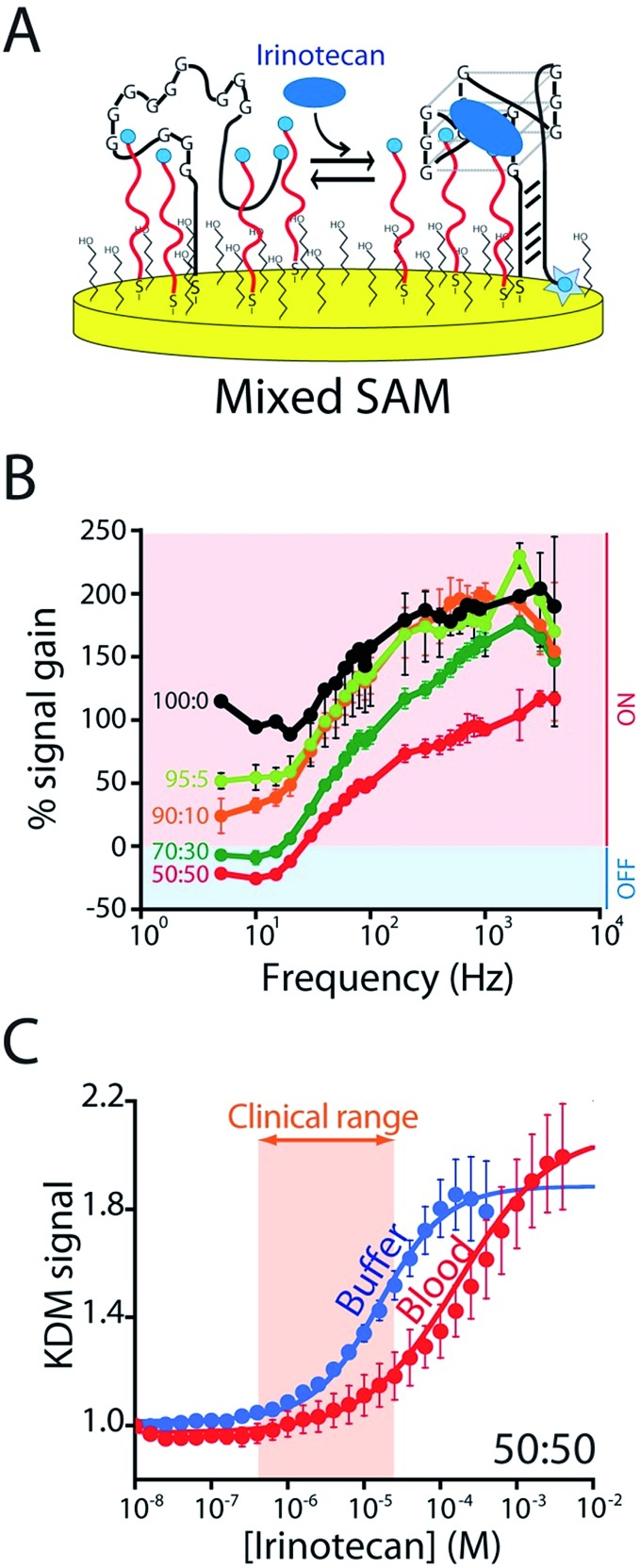
To correct the drift of seen during *in vivo* deployment we modified the E-AB sensor so that it better supports “Kinetic Differential Measurements” (KDM). (A) KDM requires that the gain of an E-AB sensor be a strong function of square wave frequency.^22^ To induce this we co-deposited the aptamer with a redox-reporter-modified linear DNA sequence that does not respond to target. (B) The signal gain (relative signal change between no target and saturating target – *i.e.*, 100 μM) of the original E-AB sensor (100 : 0 black curve) is a relatively minor function of square-wave frequency. Upon co-deposition with increasing amounts of the linear-strand (to a maximum ratio of 50 : 50, red curve) we observe increasingly strong frequency dependence, albeit with a corresponding reduction in the maximum gain. (C) A sensor fabricated using a 50 : 50 mixture of the two strands and employing KDM drift correction (here the difference in the relative signals seen at 10 and 120 Hz) responds to target over the clinically-relevant range (0.5 μM to 15 μM; 0.06 and 10 μg mL^–1^)^17^,^18^ in both buffer and in undiluted whole blood.

To enhance the frequency-dependence of the sensor's gain in support of KDM drift correction we added a second reporter-modified DNA strand to the sensor that: (1) transfers electrons more rapidly than the aptamer does and (2) does not respond to the presence of the target (Fig. S4A†). Our rational for doing so was that, at frequencies at which this “non-responsive” DNA dominates the signal (*i.e.*, at higher frequencies) the gain of the resultant sensor will be low, and at frequencies at which, instead, the aptamer dominates the signal gain will be higher (Fig. S5B†). To achieve this we co-deposited the CA32 aptamer variant and an unstructured 10-base strand comprised of a random sequence of adenines and thymines (Fig. 4A) that is known to transfer electrons at a rate of 80 s^–1^ (Fig. S4†).^23^ Per our expectations, sensors employing a 1 : 1 mixture of this sequence and the aptamer achieve sufficiently frequency-dependence gain to enable KDM (Fig. 4B). To our surprise, however, the resultant frequency dependence is so strong that the sensor's gain becomes slightly negative at low frequencies, an observation inconsistent with the expectations described above (if the currents are additive, the gain cannot go below zero). We presume this occurs due to interactions between the two sequences on the surface that alter their electron transfer kinetics. Irrespective of its origins, however, the effect supports accurate KDM drift correction. Specifically, using the signals obtained at 10 Hz (signal-off) and 120 Hz (signal-on) to perform KDM (Fig. S5†) we can easily monitor irinotecan in both buffer and whole blood (Fig. 4C) over the entire 0.5 to 15 μM (0.06 to 10 μg mL^–1^) human therapeutic range.^17^,^18^

**Fig. 5:**
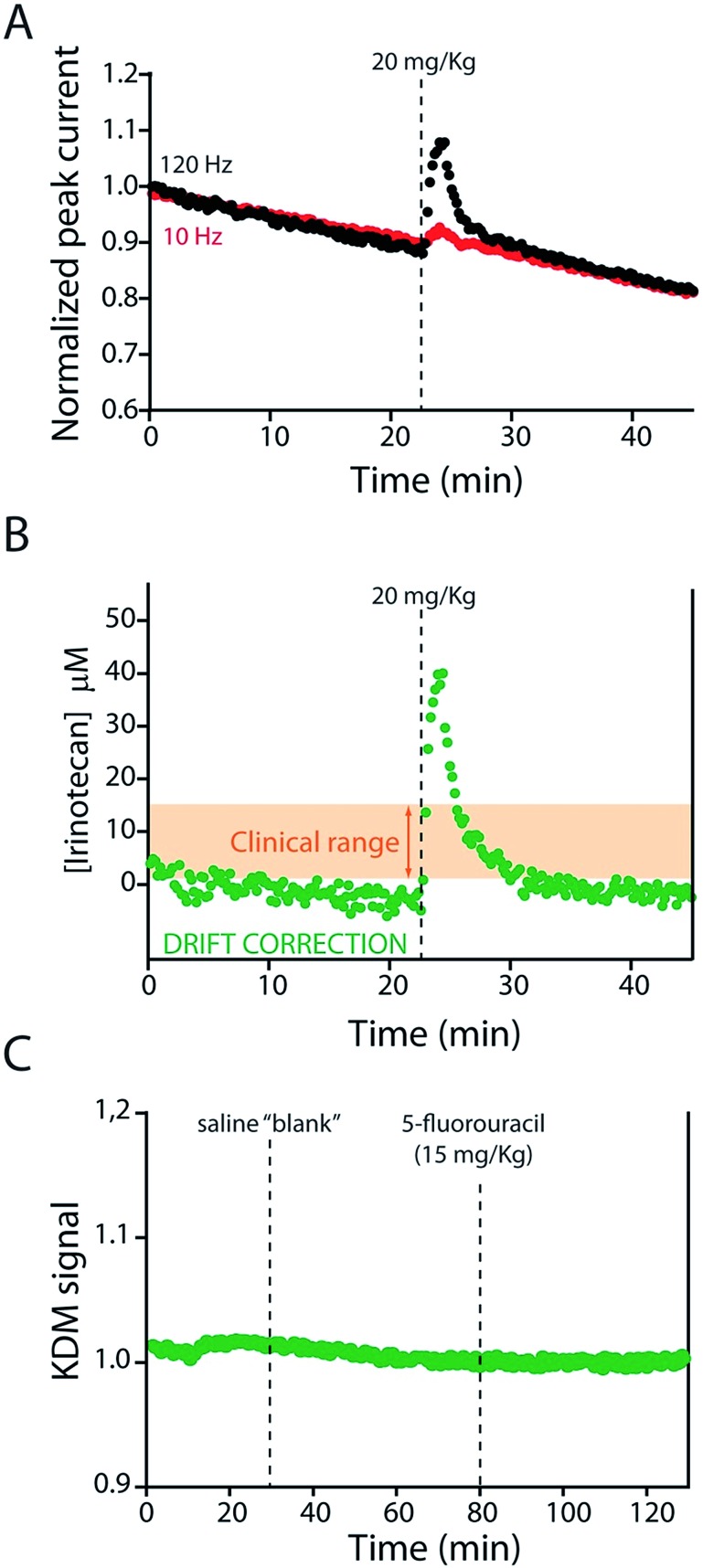
The KDM-corrected E-AB sensor supports real time, seconds-resolved measurements of plasma irinotecan levels.^9^,^22^ (A) In the absence of KDM signals collected at high (120 Hz) and low (10 Hz) frequencies both drift significantly, but because they drift in concert (B) taking their difference (KDM) produces a stable baseline. (C) As expected, control injections of either a saline “blank” or a second chemotherapeutic (5-fluorouracil, which is often co-administered with irinotecan)^31^,^32^ do not produce any measurable sensor response.

KDM-corrected indwelling E-AB sensors readily support the real-time, high frequency irinotecan measurements *in situ* in the bodies of live rats. To demonstrate this we fabricated sensors using 75 μm-diameter gold, platinum and silver wires as the working, counter and reference electrodes, respectively (Fig. 1B). We inserted the resulting sensor in the jugular vein of anesthetized Sprague-Dawley rats *via* a previously emplaced 22-gauge catheter (Fig. 1C). Testing this with a single intravenous injection (20 mg kg^–1^) of irinotecan we find that the signal observed at both 10 and 120 Hz respond to the drug, but these are also accompanied by the expected^9^ signal drift (Fig. 5A). And the gain observed at 10 Hz becomes, under these conditions, slightly positive. We are nevertheless still able to use KDM to correct the sensor's drift and recover stable baselines, thus enabling continuous, real-time measurements of the drug at therapeutically relevant concentrations (Fig. 5B).

**Fig. 6:**
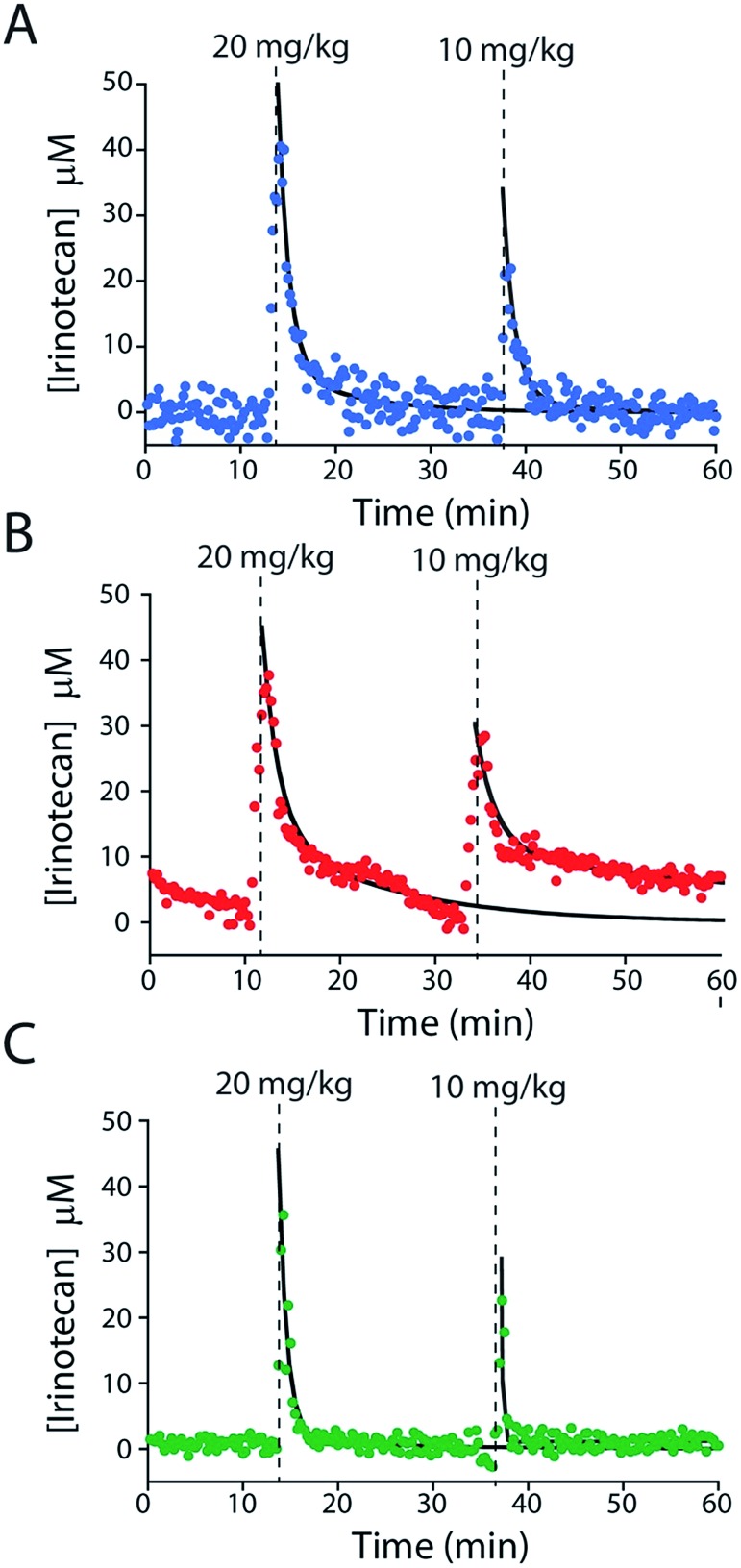
E-AB sensors support the measurements of plasma drug levels with unprecedented time resolution, providing a high-precision window into, for example, intra-subject pharmacokinetic variability. Shown are plasma irinotecan levels following multiple intravenous injections into three live rats (A–C). The black lines represent the fit of each injection dataset to a two-compartment pharmacokinetic model.

To further characterize the performance of the camptothecin sensor we used it to monitor sequential intravenous injections of irinotecan (at 10 and 20 mg kg; Fig. 6A). The resultant maximum concentrations (*C*_MAX_ = 39.8 ± 3.2 μM and 20.9 ± 2.0 μM, respectively; here and below the confidence intervals reflect standard errors calculated from the fits) and distribution rates (*α* = 0.58 ± 0.07 min and 0.48 min (fixed value, see ESI Table 2†)) are comparable to those seen in previous studies that employed ex-vivo drug-level measurements (Fig. S6A and Table S2†).^24^–^28^ In contrast, the elimination rates (*β* = 9.2 ± 1.4 min and 8.4 ± 2.2 min) we observe are more rapid than those reported previously and, thus, the resulting “areas under the curve” for the drug are reduced (Fig. S7 and Table S2†).^24^–^28^ We believe this discrepancy (Fig. S7†) occurs because the prior work used chromatographic and mass spectrometric methods to measure total drug levels (which requires removal of blood samples from the animal's body and the extraction of the total drug into buffer).^24^–^28^ E-AB sensors, in contrast, measure the free drug, which is the fraction of the drug that is pharmacologically active.^29^ And, in general, the elimination and clearance of free drug are more rapid than those of total drug as drugs that interact strongly with plasma proteins tend to clear more slowly than those that do not.^30^

E-AB-derived measurements of irinotecan pharmacokinetics represent a significant advance over prior pharmacokinetic studies of the camptothecins.^24^–^28^ For example, the 20 s temporal resolution of our measurements (defined by the time required to take the two square wave scans necessary to perform KDM) is at least an order of magnitude better than that of the most highly time resolved prior study.^24^–^28^ Moreover, all prior studies reported plasma level measurements averaged over multiple animals, thus eliminating their ability to explore subject-to-subject pharmacokinetic variability. The present E-AB-derived measurement parameters, in contrast, provide 300 time points per hour in each animal, and thus determine the pharmacokinetics of individuals with exceptional precision. Because the excretion phase of irinotecan exhibits significant inter-patient variability (due to drug–drug interactions, variations in health status, and pharmacogenetics),^17^,^18^,^33^,^34^ this latter point is likely of clinical significance. To illustrate our ability to measure such variability we performed sequential 10 and 20 mg kg^–1^ irinotecan injections in three rats (Fig. 6). The resulting measurements reveal only small (∼10 to 20%) variation in either *C*_MAX_ or the rate of the distribution phase (Fig. S6B and C, and Table S2†). In contrast, however, the rate of drug excretion and its clearance values vary many fold from individual to individual. As all of the animals we employed in these experiments were healthy male rats these pharmacokinetic differences arose solely due to metabolic variability between the animals.

**Fig. 7:**
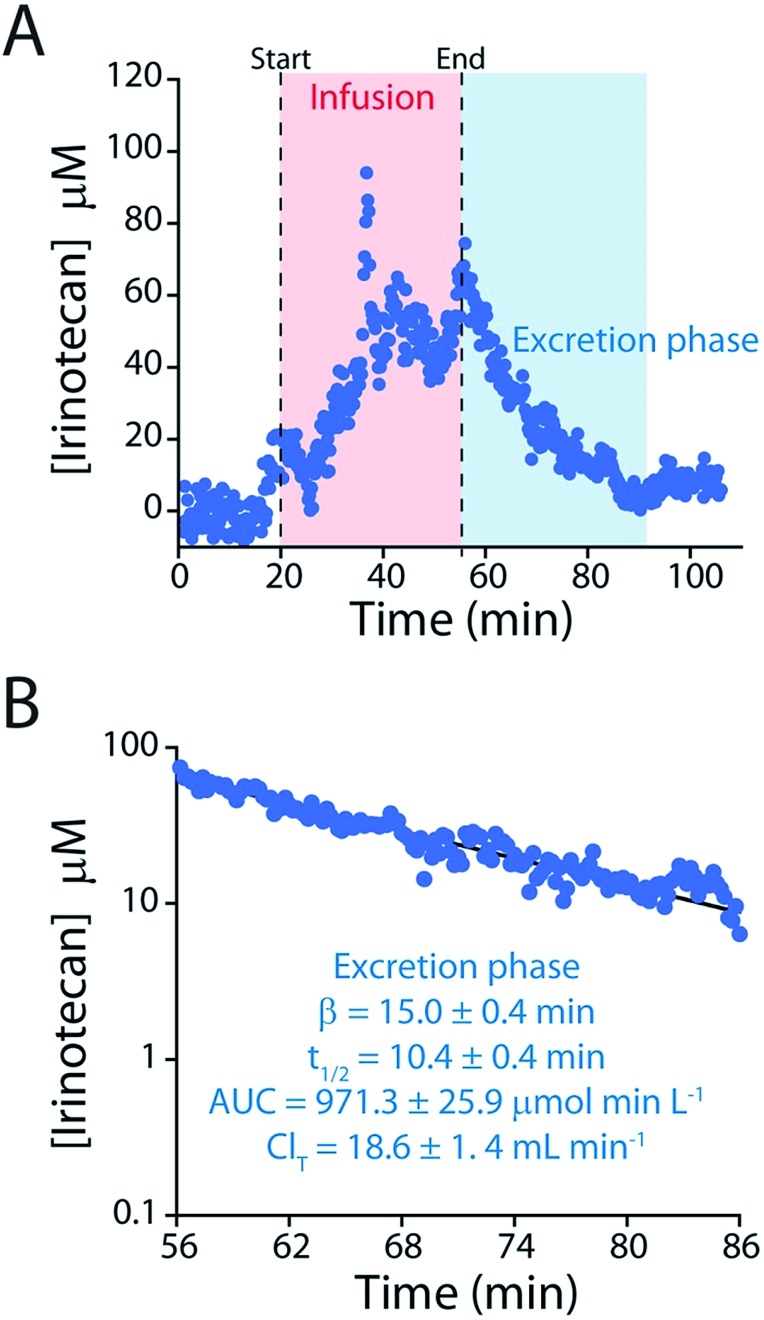
(A) To determine irinotecan's pharmacokinetics with more precision we performed an intravenous injection at a much higher dose (60 mg kg^–1^). (B) The higher peak concentrations reached in this experiment lead to longer measurement runs, in turn improving the precision of our estimates of the relevant pharmacokinetic parameters.

The elimination rate and clearance of irinotecan are the pharmacokinetic parameters used to determine its optimized, personalized dosing during chemotherapy.^17^,^18^,^33^,^34^ To measure these parameters with greater precision we administered a large dose (60 mg kg^–1^) of the drug over a longer period. The higher plasma concentrations this produces lead, in turn, to a longer measurement period (after delivery ceases) before the sensor's limit of detection is reached (Fig. 7). The ∼150 plasma drug measurements we thus achieve in a single pharmacokinetic profile produces estimates of the drug's elimination half-life (10.4 ± 0.4 min) and clearance (18.6 ± 1.4 mL min^–1^) that are far more precise than those produced in prior, *ex vivo* studies, which typically achieve less than a dozen measurements per profile,^24^–^28^ much less the two measurements used in typical “peaks-and-troughs” clinical measurements.

## Conclusions

Here we describe an indwelling E-AB sensor supporting the seconds-resolved measurement of the anticancer drug irinotecan *in situ* in the living body over the course of hours. Design of the sensor required the reengineering of a parent aptamer to support high-gain E-AB signaling and the development of a novel method to ensure sufficient frequency-dependent signal gain to support KDM-based drift correction. Using the resulting sensors we measured plasma irinotecan levels with micromolar concentration resolution and seconds temporal resolution, with the latter representing an orders of magnitude improvement over that of prior studies. The resulting measurements define the pharmacokinetics of irinotecan of individual animals, providing an unprecedented high precision view of the drug's inter-subject pharmacokinetic variability.

E-AB sensors are a platform technology that supports the high frequency, real-time measurement of specific molecules (irrespective of their chemical reactivity) *in situ* in the living body. When coupled with the platform's convenience and precision this versatility provides significant opportunities to improve drug dosing. As noted above, for example, irinotecan suffers from significant inter-patient metabolic variability,^35^,^36^ leading to toxicity and increasing side effects.^17^,^34^ But because current methods for measuring plasma drug levels are slow and cumbersome,^37^ the FDA has invoked pharmacogenetic estimates of metabolism as the primary means of reducing the risk associated with this variation.^35^ In this light, the ease with which E-AB sensors provide high precision, patient-specific measurements of drug elimination (as opposed to indirect estimates), suggests the platform could provide a valuable adjunct to chemotherapeutic treatment.

In addition to improving the precision and accuracy of personalized dose determination, E-AB-derived measurements may also support a new paradigm for personalized drug delivery. Specifically, we have recently used the real-time concentration information provided by E-AB sensors to inform closed-loop feedback controlled drug delivery.^5^ In this the rate of drug administered is optimized multiple times a minute, enabling the maintenance of plasma drug concentrations at a pre-defined value with precision of better than 20% despite ∼3-fold hour-to-hour changes in drug pharmacokinetics. This approach to drug delivery provides an unprecedented means of overcoming pharmacokinetic variability, improving the overall efficacy and safety of treatment. Given this, drug-detecting E-AB sensors could prove a powerful new tool in the clinician's arsenal.

## Statement of contributions

A. I., N. A. C., T. E. K., and K. W. P. conceived the experiments. A. I., N. A. C. and A. C. developed the E-AB sensors and carried out experiments *in vitro*. A. I., N. A. C. and K. L. P. carried out the experiments *in vivo*. K. L. P. performed all the animal surgeries. T. E. K. created the animal study protocol. K. L. P. and T. E. K. supervised and enforced approved animal protocols. A. I., N. A. C., T. E. K., and K. W. P. wrote the paper. All authors have given approval to the final version of the manuscript.

## Conflicts of interest

One author (K. W. P.) has a financial interest in and serves on the scientific advisory boards of two companies attempting to commercialize E-AB sensors. A. I., N. A. C. and K. W. P. have filed a provisional patent based on the work presented in this paper.

## Supplementary Material

Supplementary information
