# Supplementary material for: Seconds-resolved pharmacokinetic measurements of the chemotherapeutic irinotecan in situ in the living body
Source: Chem Sci. 2019 Jul 22;10(35):8164–70. doi: 10.1039/c9sc01495k (PMC6788505; doi:10.1039/c9sc01495k)
Supplement: Supplementary file 1 [file SC-010-C9SC01495K-s001.pdf]

Electronic Supplementary Material (ESI) for Chemical Science.
This journal is © The Royal Society of Chemistry 2019

Seconds-resolved pharmacokinetic measurements of the chemotherapeutic irinotecan in situ in the living body

Andrea Idili,[a,b] Netzahualcóyotl Arroyo-Currás,[c] Kyle L. Ploense,[a,b,d] Andrew T. Csordas,[a,b] Masayasu Kuwahara,[e] Tod E. Kippin[d,f,g] and Kevin W. Plaxco[a,b,]*

[a]Department of Chemistry and Biochemistry, [b]Center for Bioengineering, University of California, Santa Barbara; Santa Barbara, CA 93106 USA

[c]Department of Pharmacology and Molecular Sciences, Johns Hopkins School of Medicine, Baltimore, Maryland 21205, United States

[d]Department of Psychological and Brain Sciences; University of California, Santa Barbara; Santa Barbara, CA 93106 USA

[e] Graduate School of Integrated Basic Sciences, Nihon University, 3-25-40 Sakurajosui, Setagaya-ku, Tokyo 156-8550, Japan

[f]Department of Molecular Cellular and Developmental Biology; University of California, Santa Barbara; Santa Barbara, CA 93106 USA.

[g]Department of Neuroscience Research Institute; University of California, Santa Barbara; Santa Barbara, CA 93106 USA.

Supplementary Information

MATERIALS AND METHODS

Chemical reagents and materials. Reagent-grade chemicals, including sodium hydroxide, sulfuric acid, 6-mercapto-1-hexanol, sodium chloride, ethanol, potassium chloride, potassium phosphate dibasic, sodium phosphate monobasic, tris(2-carboxyethyl)-phosphine hydrochloride (TCEP), hydrochloride tris[hydroxymethyl]- aminomethane hydrochloride, magnesium chloride, dimethyl sulfoxide (DMSO), ethylenediaminetetraacetic acid (EDTA) and 5-fluorouracil (USP grade) (all from Sigma-Aldrich, St. Louis, MO, USA) were used as received. irinotecan hydrochloride (USP grade) was purchased from Fischer Scientific (Waltham, MA, USA) and used as received.

Catheters (22 G) and 1 mL syringes were purchased from Becton Dickinson (Franklin Lakes, NJ). Polytetrafluoroethylene-insulated gold, platinum, and silver wires (75 µm diameter) were purchased from A-M systems. To employ the silver wires as reference electrodes they were immersed in concentrated sodium hypochlorite (commercial bleach) overnight to form a silver chloride film. Heat-shrink polytetrafluoroethylene insulation (Sub-Lite-Wall, 0.02, 0.005, 0.003 ± 0.001 in, blackopaque, Lot No. 17747112-3), used to electrically insulate the wires, was purchased from ZEUS (Branchburg Township, CA). Custom-made, open ended, mesh-covered three channel connector cables to fabricate in vivo probes were purchased from PlasticsOne (Roanoke, VA).

Oligonucleotides. HPLC purified oligonucleotides were purchased from Biosearch Technologies (Novato, CA, USA). For sensor fabrication the aptamer variants were purchased modified with a thiol-C_6-SS group at its 3' end, and a methylene blue attached by a six-carbon linker to an amine at its 5' end. The oligonucleotides were dissolved in buffer (100 mM Tris buffer, 10 mM $MgCl_2$, pH 7.8) at a concentration of 100 µM and then aliquoted and stored at -20 °C. The final concentrations of the oligonucleotides were confirmed using a Beckman Coulter DU 800 Spectrophotometer (Männedorf, Switzerland) using a 100 µL quartz cuvette and measuring the relative absorbance at 260 nm.

The aptamer sequences we employed were a re-engineered version of a previously reported aptamer against this target:[1]

CA40: 5'-**ACGCT CCGGA CTT**GG GTGGG TGGGT TGGG**G TACGG TGCGT**-3'

CA40_1MM: 5'-**ACGCT CCGGA CTT**GG GTGGG TGGGT TGGG**G TACGG TGTGT**-3'

CA40_2MM: 5'-**ACGCT CTGGA CTT**GG GTGGG TGGGT TGGG**G TACGG TGTGT**-3'

CA36: 5'-**GCTCC GGACT T**GGGT GGGTG GGTTG GG**GTA CGGTG C**-3'

CA32: 5'-**TCCGG ACTT**G GGTGG GTGGG TTGGG **GTACG GT**-3'

CA28: 5'-**CGGAC TT**GGG TGGGT GGGTT GGG**GT ACG**-3'

CA16: 5'- GGGTGGGTGGGTTGGG -3'

For all the sequences above the bases in bold represent the stem portion and the underlined bases represent the G-quadruplex binding pocket for the aptamer variants.

The linear probe was modified with a thiol-C_6-SS group at its 3' end and a methylene blue at its 5' and was of the following sequence:

5'-ATTATTTTT-3'

The unmodified CA40 aptamer variant used for the fluorescence experiments had the following sequence:

CA40: 5'-**ACGCT CCGGA CTT**GG GTGGG TGGGT TGGG**G TACGG TGCGT**-3'

Fluorescence Experiments. The fluorescence data (Figures 2B and S1) was obtained using a Varian Cary Eclipse Fluorometer with excitation at 370 (±5) nm and acquisition between 400 (±5) nm and 650 (±5) nm at a temperature of 25°C, and using a total volume of 800 µl in a quartz cuvette. Irinotecan was dissolved in DMSO to reach a concentration of 100 µM and, then, diluted in the working buffer (NaCl 137 mM, KCl 2.7 mM, Na_2HPO_4 10 mM, KH_2PO_4 1.8 mM at pH 7.4) at a final concentration of 1 µM. We recorded the fluorescence signal of irinotecan in the absence of CA40 until a stable signal was obtained. We then added unmodified CA40 aptamer variant (stock solution 100 µM) at various concentrations and, after 15 min, fluorescence spectra were recorded (Figure S1A). The fluorescence intensity at 443 nm was used to generate binding curves (Figures 2B and S1B) and fitted using a single-site binding model:[2]

$$F^{[CA40]}_{IPT} = F^{0}_{IPT} + \left(\frac{[CA40]\left(F^{CA40MAX}_{IPT} - F^{0}_{IPT}\right)}{[CA40] + K_D}\right) \quad (1)$$

Where [CA40] is the concentration of aptamer variant, $F^{[CA40]}_{IPT}$ is the irinotecan fluorescence signal in the presence of different concentrations of CA40, F^{0}_{IPT} is the background fluorescence of irinotecan, $F^{CA40MAX}_{IPT}$ is the irinotecan fluorescence signal in the presence of saturating concentrations of CA40, and K_D is the dissociation constant of the binding process. Using the estimated values for F^{0}_{IPT} and $\left(F^{CA40MAX}_{IPT} - F^{0}_{IPT}\right)$ we normalized the raw fluorescence data using the following equation:

$$F^{NORM}_{IPT} = 1 + \left(\frac{F^{[CA40]}_{IPT} - F^{0}_{IPT}}{\left(F^{CA40MAX}_{IPT} - F^{0}_{IPT}\right)}\right) \quad (2)$$

In-vitro Sensors

Electrode Polishing and Cleaning. The E-AB sensors employed in-vitro (Figures 2C, 2D, 3B, 3C, 4 and Figures S3, S4, S5) were fabricated using established approaches.[3] Briefly, E-AB sensors were fabricated on rod gold disk electrodes (3.0 mm diameter, BAS, West Lafayette, IN, USA). The disk electrodes were prepared by polishing on a microcloth pad soaked with a 1 µm diamond suspension slurry (MetaDi, Buehler, Lake Bluff, IL, USA) and then with a 0.05 µm alumina powder aqueous suspension. Each polishing step is followed by sonication of the electrodes in a solution 1:1 water/ethanol for 5 min. The electrodes were then electrochemically cleaned following this procedure: a) The electrodes are placed in a 0.5 M NaOH solution and through cyclic voltammetry 1000-2000 scans are performed using a potential between −0.4 and −1.35 V versus Ag/AgCl at a scan rate of 2 V s^{-1}; b) The electrodes were moved to a 0.5 M H_2SO_4 solution and using chronoamperometry an oxidizing potential of 2 V was applied for 5 s. After a reducing potential of −0.35 V was then applied for 10 s; c) Using cyclic voltammetry we cycled the electrodes rapidly (4 V s^{-1}) in the same solution between −0.35 and 1.5 V for 10 scans followed by 2 cycles recorded at 0.1 V s^{-1} using the same potential window.

Electrode functionalization. For the E-AB sensors functionalized with a classic monolayer composed by a single DNA probe (Figures 2C, 2D, 3B and 3C and Figures S3 and S4), we first reduced the DNA probe (100 µM) by treating it for 1 h in a solution of 10 mM tris(2-carboxyethyl)-phosphine hydrochloride (TCEP) at room temperature in the dark. This was then dissolved in

"assembling buffer" (10 mM Na_2HPO_4 with 1 M NaCl and 1 mM $MgCl_2$ at pH 7.3) at a final concentration of 500 nM. The electrochemically cleaned gold electrodes were then immersed in 200 µL of this solution for 1 h in the dark. Following this the electrode surface was rinsed with distilled water and incubated overnight at 4°C in assembling buffer containing 5 mM 6-mercaptohexanol, followed by a further rinse with distilled water before use. For the E-AB sensors functionalized with a mixed monolayer formed by CA32 aptamer and the linear probe (Figures 4 and S5), we first reduced the two DNA probes (100 µM) by treating them for 1 h in a solution of 10 mM tris(2-carboxyethyl)-phosphine hydrochloride (TCEP) at room temperature in the dark. These were then dissolved in buffer (10 mM Na_2HPO_4 with 1 M NaCl and 1 mM $MgCl_2$ at pH 7.3) using different ratio of the two DNA probes. The total oligonucleotide concentration was kept constant at 500 nM to maintain a constant DNA probes packing density. The electrochemically cleaned gold electrodes were then immersed in 200 µL of this solution composed of two DNA probes for 1 h in the dark. Following this step, the electrode surface was rinsed with distilled water and incubated overnight at 4°C in assembling buffer containing 5 mM 6-mercaptohexanol, followed by a further rinse with distilled water before use.

In-vivo Sensors

Electrode fabrication. The E-AB sensors employed in-vivo (Figures 5, 6, 7, S6 and S7) were fabricated as described in previous reports.[4,5] Segments of pure gold (7.75 cm in length), platinum (7.25 cm), and silver (6.75 cm) wire, were cut to make sensors. The insulation at both ends of these wires, about 1 cm, was removed using a surgical blade to allow electrical contact. These were then soldered each to one of the three ends of a connector cable using 60% tin/40% lead rosin-core solder (0.8 mm diameter) and then attached together by applying heat to shrinkable tubing around the body of the wires, except for a small window of about 5 mm at the edge of each wire. The wires were attached in a layered fashion, with the gold wire being insulated alone first, then both gold and platinum wires together, and finally all three wires together. The purpose of this three-layer-thick insulation was to give mechanical strength to the body of the malleable probe. To prevent electrical shorts between wires, different lengths were used for each wire as described above. The sensor window (i.e., the region devoid of insulation) in the gold wire was cut to approximately 3 mm in length. To increase the surface area of the gold working electrodes (to obtain larger peak currents) the sensor surface was roughened electrochemically via immersion in 0.5 M sulfuric acid followed by stepping the potential between $E_{initial}$ = 0.0 V to E_{high} = 2.0 V vs Ag/AgCl, back and forth, for 16 000 pulses.[4] Each potential step was of 20 ms duration with no wait time between pulses.

Electrode functionalization. The E-AB sensors employed in-vivo were functionalized with a mixed monolayer formed by CA32 aptamer and the linear probe (Figures 5, 6, 7, S6 and S7) . We first reduced the two DNA probes (100 µM) by treating them for 1 h in a solution of 10 mM tris(2-carboxyethyl)-phosphine hydrochloride (TCEP) at room temperature in the dark. These were then dissolved in "assembling buffer" (10 mM Na_2HPO_4 with 1 M NaCl and 1 mM $MgCl_2$ at pH 7.3) employing a 1:1 mixture of the two DNA probes (i.e. 250 nM for each DNA probe) for a total oligonucleotides concentration of 500 nM. The electrochemically roughened gold electrode was rinsed in deionized water before being immersed in 200 µL of this solution composed by two DNA probes for 1 h in the dark. Following this the electrode surface was rinsed with distilled water and incubated overnight at 25°C in assembling buffer containing 5 mM 6-mercaptohexanol, followed by a further rinse with distilled water before use.

In-vitro measurements. Electrochemical measurements were performed at room temperature using a CHI660D potentiostat with a CHI684 Multiplexer (CH Instruments, Austin, TX) and a standard three-electrode cell containing a platinum counter electrode and a Ag/AgCl (3 M KCl) reference electrode. Square Wave Voltammetry (SWV) was performed using a potential window of -0.1 to -0.4 V, a potential step of 1 mV and an amplitude of 50 mV.

Titration curves. Experimental titration curves (Figure 2C, 3B, 4B, S3, S6A and S6B) were performed in 10 mL of working buffer (137 mM NaCl, KCl 2.7 mM, Na_2HPO_4 10 mM, KH_2PO_4 1.8 mM at pH 7.3) using three E-AB sensors modified with the oligonucleotide probe and using a SWV frequency of 10 and 120 Hz. Initially, in absence of irinotecan, we performed a preliminary treatment by interrogating the electrodes with 30-60 scans until a stable current peaks were obtained. Once the sensor's signal was stable increasing concentrations of the irinotecan was added and the sensors were interrogated after 10 min. The electrochemical signal (peak current) of each sensors was plotted in function of irinotecan concentrations and then it was fitted using a single-site binding mechanism equation in Kaleidagraph (Synergy Software):

$$C_{Raw}^{[Irinotecan]} = C_{Raw}^{0} + \left(\frac{[Irinotecan]\left(C_{MAX}^{[Irinotecan]} - C_{Raw}^{0}\right)}{[Irinotecan] + K_D} \right) \quad (3)$$

Where [Irinotecan] is the irinotecan concentration, $C_{Raw}^{[Irinotecan]}$ is signal current in the presence of different concentrations of irinotecan, C_{Raw}^{0} is the background current seen in the absence of the irinotecan, $C_{MAX}^{[Irinotecan]}$ is the current signal seen at saturating concentrations of irinotecan, and K_D *i*s the dissociation constant of the surface-bound aptamer. Using the values estimated from the previous fitting procedure we converted the raw signal current in signal change % ($C_{\%}$) using the following equation:

$$C_{\%} = \left(\frac{C_{Raw}^{[Irinotecan]} - C_{Raw}^{0}}{C_{Raw}^{0}} \right) \cdot 100 \quad (4)$$

The binding curves performed in whole blood (Figure 2C, 3C, 4B, S6C and S6D) were conducted following the previous described experimental approach but using a closed-loop system with a continuous flow of whole blood (total volume of 20 mL at 1 mL/s) through a circulator pump.

Sensor equilibration time. We determined the sensor's equilibration time (Figure 2D) using the above experimental approach and interrogating the sensor every 5 s in working buffer and using a SWV frequency of 500 Hz. After we achieved a stable current baseline (5 min) we added to the solution different concentrations of irinotecan (from 1 µM to 1 mM) and we monitored the voltammetric signal for over 5 min. Then we moved the sensor in simple working buffer without irinotecan and we collected the voltammetric signal for over 5 min. The observed signal change was fitted to a single exponential decay in Kaleidagraph (Synergy Software) to obtain the equilibration time constant of the sensor.

Square wave voltammetry frequency vs signal change (%) plot. We estimated the dependence of E-AB sensors's signal change (%) on the square wave voltammetry frequencies (Figures 4A, S4 and S5) interrogating the sensors using various frequencies (from 5 Hz to 4000 Hz) in absence and in presence of a saturating amount of irinotecan (1 mM and 100 µM, and an incubation time of 10 min). The collected peaks current, in absence and in presence of a saturating amount of irinotecan (1 mM or 100 µM), were converted in signal change % using equation (4). Then the estimated signal change (%) was plotted in function of the relative square wave frequency using Kaleidagraph (Synergy Software).

In-vivo measurements. All in vivo measurements were performed using a three-electrode setup in which the counter electrodes were made of platinum wire and the reference electrodes were a silver wire coated with a silver chloride film as described above. The measurements carried out in vivo were recorded using a handheld potentiostat from CH Instruments (Model 1242 B). Square Wave

Voltammetry (SWV) was performed using a potential window of -0.1 to -0.45 V, a potential step of 0.001 V and 0.05 V amplitude.

For in vivo measurements (Figures 5, 6 and 7) a 30 min sensor baseline was established before the drug infusion. A 3 mL syringe filled with the target drug was connected to the sensor-free catheter (placed in the jugular opposite that in which the sensor is emplaced) and placed in a motorized syringe pump (KDS 200, KD Scientific Inc., Holliston, MA, USA). After establishing a stable baseline, the drug was infused through this catheter at a rate of 0.6 mL/min using irinotecan at 15 mg/mL or 5-fluorouracil at 10 mg/mL. After drug infusion, recordings were taken for up to 1 h before the next infusion. The real-time plotting and analysis of voltammetric data were carried out with the help of a script written in Python.

Animals. In vivo measurements were performed in male and female Sprague-Dawley rats (4−5 months old) purchased from Charles River Laboratories (Santa Cruz, CA), weighing between 300 and 500 g. All animals were pair housed in a standard light cycle room (08:00 on, 20:00 off) and allowed ad libitum access to food and water.

Surgery. For in vivo measurements rats were induced under 5% isoflurane anesthesia in a Plexiglas anesthesia chamber. The rats were then maintained on 2−3% isoflurane gas for the duration of the experiment. While anesthetized, surgery was performed to emplace both an infusion line and the E-AB sensor within the jugular veins. Briefly, the area above each jugular vein was shaved and cleaned with betadine and 70% ethanol. A small incision was made above each vein, then each vein was isolated. A small hole was cut into each vein with spring-loaded microscissors. The silastic catheter inserted into the left jugular vein was constructed with a bent steel cannula with a screw-type connector (Plastics One, Roanoke, VA) and silastic tubing (11 cm, i.d. 0.64 mm, o.d. 1.19 mm, Dow Corning, Midland, MI).[4,5] The EAB sensor was constructed as previously mentioned and inserted into the right jugular vein. Both the E-AB sensor and the infusion line were tied into place with sterile 6−0 silk suture (Fine Science Tools, Foster City, CA), then 30 units of heparin. Once EAB measurements were concluded, the rats were overdosed on 5% isoflurane gas until breathing and heartbeat ceased, and were decapitated with a guillotine.

Pharmacokinetic Analysis. Plasma irinotecan concentration-time curves (Figures 6, S6 and Table S2) were fitted to a bi-exponential equation describing a two-compartment model:

$$[Irinotecan] = Ae^{-t/\alpha} + Be^{-t/\beta} \qquad (5)$$

Where A and B are amplitudes, and α and β are the half-lives for distribution and elimination, respectively. To improve the precision of the fitting we used a time range of 20 min for the pharmacokinetic curves obtained using an intravenous dose of 20 mg/kg of drug, and a time range of 10 min for the curves obtained using an intravenous dose of 10 mg/kg of irinotecan. Moreover, to improve the precision of the fitting equation we fixed α and β for the rat 1 and rat 3 for the curves obtained using an intravenous dose of 10 mg/kg of irinotecan. The peak concentration (C_{MAX}) was directly obtained from the experimental values of each irinotecan concentration-time curves. The area under the curve (AUC) was calculated using the parameters A, B, α and β, because integrating the equation 5 from 0 to infinity, it holds:[7]

$$AUC = \frac{A}{\left(\frac{1}{1/\alpha}\right)} + \frac{B}{\left(\frac{1}{1/\beta}\right)} \qquad (6)$$

The clearance (Cl_T) was calculated by Equation 6:

$$Cl_T = \frac{Dose}{AUC} \qquad (7)$$

The errors for C_{MAX}, AUC and Cl_T are propagated from the errors associated to the kinetic parameters A, B, α and β.

NUPACK Simulations. We used NUPACK (http://www.nupack.org/) to predict the folding free energies of the stem portion for the CA40 aptamer variants (Figure S2).[8] The aptamer sequences were analyzed by the software using the following parameters: (a) temperature: 25 °C; (b) number of strand species: 1; (c) maximum complex size: 4; (d) oligo concentration = 500 nM; in advanced options; (e) [Na+] = 0.15 M, [Mg++] = 0 M; (f) dangle treatment: some.

SUPPORTING FIGURES

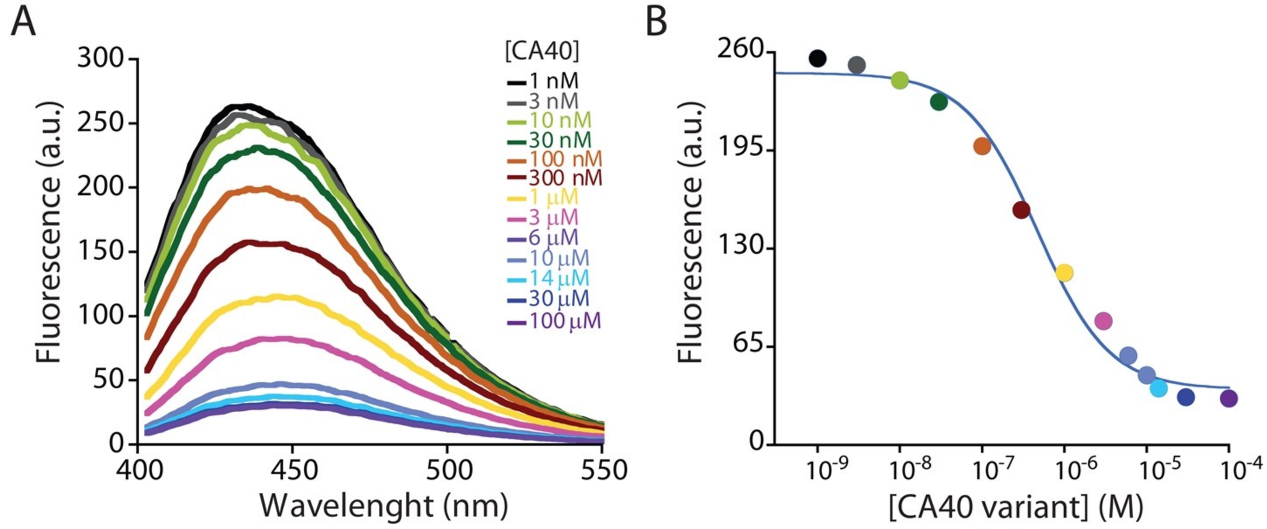

Figure S1. Exploiting the intrinsic fluorescence of irinotecan we have characterized here the affinity of the CA40 aptamer variant. (A) Fluorescence spectra collected as 1 µM irinotecan is titrated with increasing concentrations of the aptamer in working buffer (excitation at 370 nm). (B) The resultant binding curve produces a K_D of 475 ± 10 nM.

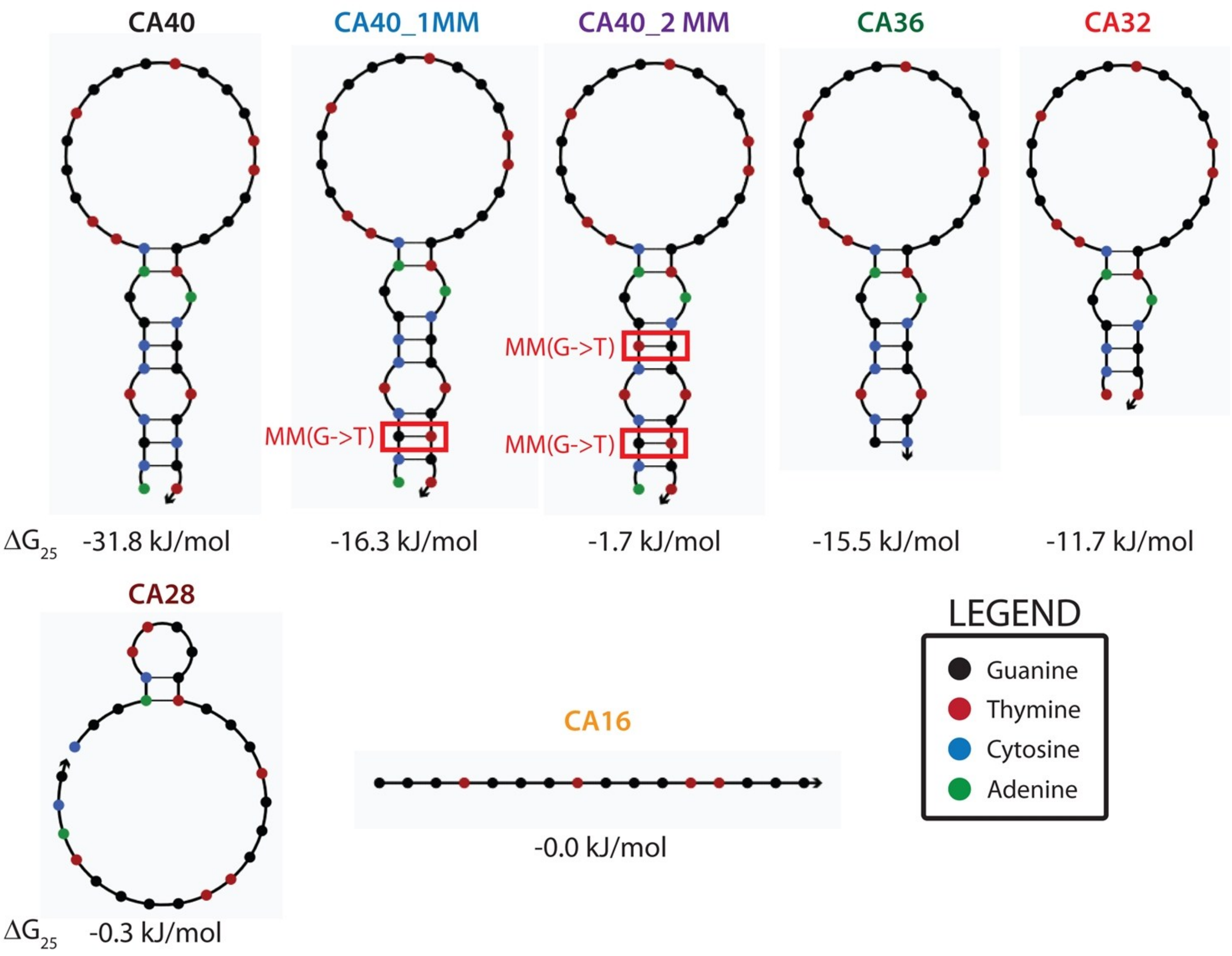

Figure S2. Shown are the secondary structures and stabilities for the aptamer variants employed here as predicted by the nucleic acid folding predictor NUPACK.[8]

Table S1. Binding affinity (K_D) and signal gain for the E-AB sensors in buffer and whole blood.

Aptamer variant	BUFFER		BLOOD	
	K_D (μM)	Signal gain (%)	K_D (μM)	Signal gain (%)
CA40	126 ± 24	84 ± 4	291 ± 15	15 ± 1
CA40_1MM	38 ± 11	119 ± 8	110 ± 22	31 ± 2
CA40_2 MM	134 ± 35	258 ± 16	130 ± 16	66 ± 2
CA36	102 ± 37	105 ± 10	290 ± 22	48 ± 1
CA32	58 ± 17	212 ± 14	331 ± 57	118 ± 6
CA28	254 ± 48	755 ± 38	850*	418 ± 12
CA16	145 ± 32	412 ± 22	850*	462 ± 5

*For the variant CA28 and CA16 the values of K_D were fixed to improve the precision of the fitting procedure (see materials and methods).

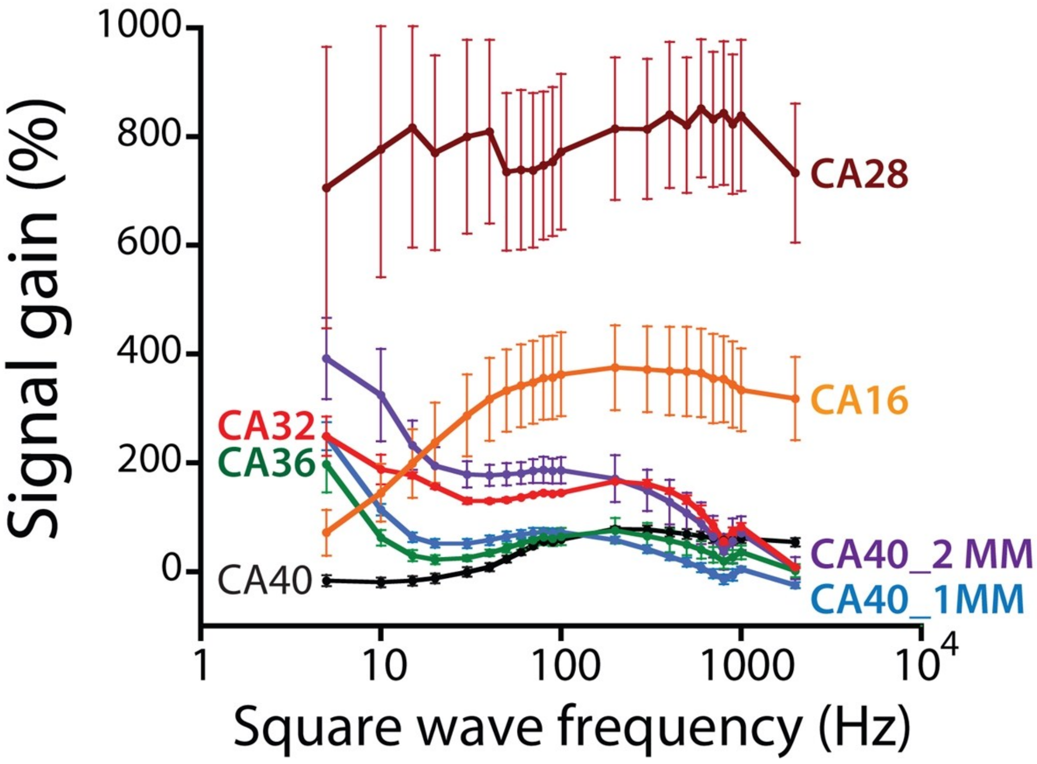

Figure S3. The signal gain (relative change in signal upon the addition of saturating target) of E-AB sensors is typically a strong function of the square wave frequency employed in its interrogation. Enough so that the gain can be either positive (signal-on behavior) or negative (signal-off) depending on the frequency employed and the electron transfer rates of the free and target-bound states of the aptamer.[9] Historically we have used this effect to remove the drift seen when E-AB sensors are deployed in vivo via a technique termed kinetic differential measurements (KDM). Uniquely in our experience, however, the gain of our camptothecin-detecting E-AB sensors is only a weak function of square-wave frequency, precluding the use of KDM. The experiments presented here were performed using 0 and 1 mM irinotecan in buffer. Errors correspond to the standard deviations observed across three independently fabricated and tested sensors.

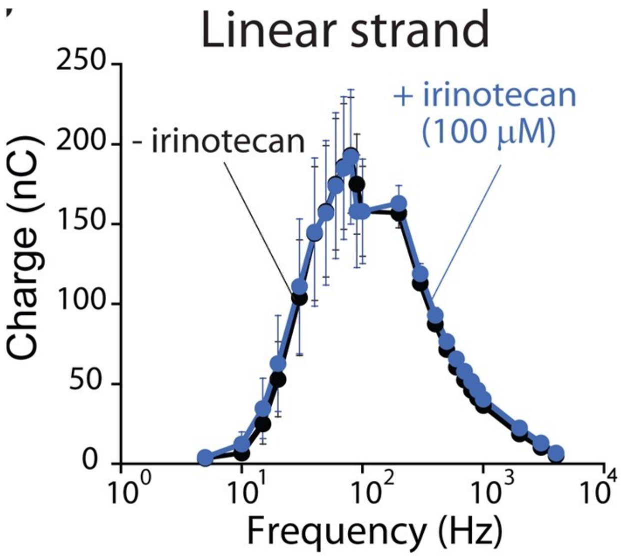

Figure S4. A methylene-blue-modified, unstructured linear DNA strand does not respond to irinotecan when employed in an E-AB sensor lacking the irinotecan-binding aptamer. Errors correspond to the standard deviations observed across three independently fabricated sensors.

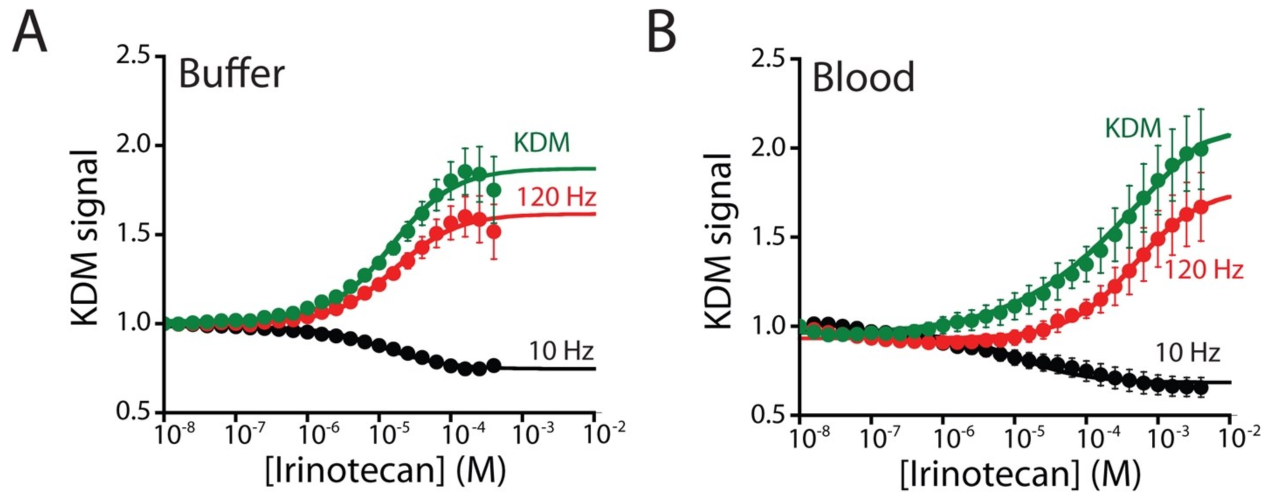

Figure S5. The binding data for the E-AB sensor collected in vitro in either (A) buffer or (B) flowing whole blood. In buffer we observe a K_D = 16.0 ± 1.3 μM for the KDM signal. In whole blood this rises to 191.2 ± 23.4 μM. Error bars correspond to the standard deviations observed across three independently fabricated and tested sensors

Table S2. The pharmacokinetic parameters associated with sequential intravenous injections of irinotecan in three Sprague– Dawley rats

Rat	Injection n.	A (μM)	α (min)	B (μM)	β (min)	C_{MAX} (μM)	AUC (μmol•min•L^{-1})	Cl_T (mL•min^{-1})
1	1	39.8 ± 3.2	0.58 ± 0.07	8.9 ± 1.1	9.2 ± 1.4	39.8 ± 3.2	105.0 ± 11.7	172.1 ± 19.2
1	2	18.6 ± 2.0	0.48*	8.4 ± 2.2	8.4 ± 2.2	20.9 ± 2.0	79.5 ± 7.1	113.7 ± 10.2
2	1	33.6 ± 2.4	0.82 ± 0.10	15.1 ± 1.0	11.6 ± 1.0	35.7 ± 2.4	202.7 ± 8.9	89.2 ± 3.9
2	2	26.6 ± 2.6	0.58 ± 0.25	11.3 ± 0.8	49.0 ± 23.3	27.9 ± 2.6	569.1 ± 20.3	15.9 ± 0.8
3	1	30.4 ± 1.7	0.65 ± 0.07	1.9 ± 0.9	13.4 ± 9.7	35.6 ± 1.7	45.2 ± 5.8	400.0 ± 51.3
3	2	22.8 ± 1.6	0.38 ± 0.06	1.3 ± 0.7	8.0*	22.6 ± 1.6	19.1 ± 9.4	473.3 ± 232.9

*For RAT 1 and RAT 3 the values of α and β were fixed to improve the precision of the pharmacokinetic analysis (see materials and methods).

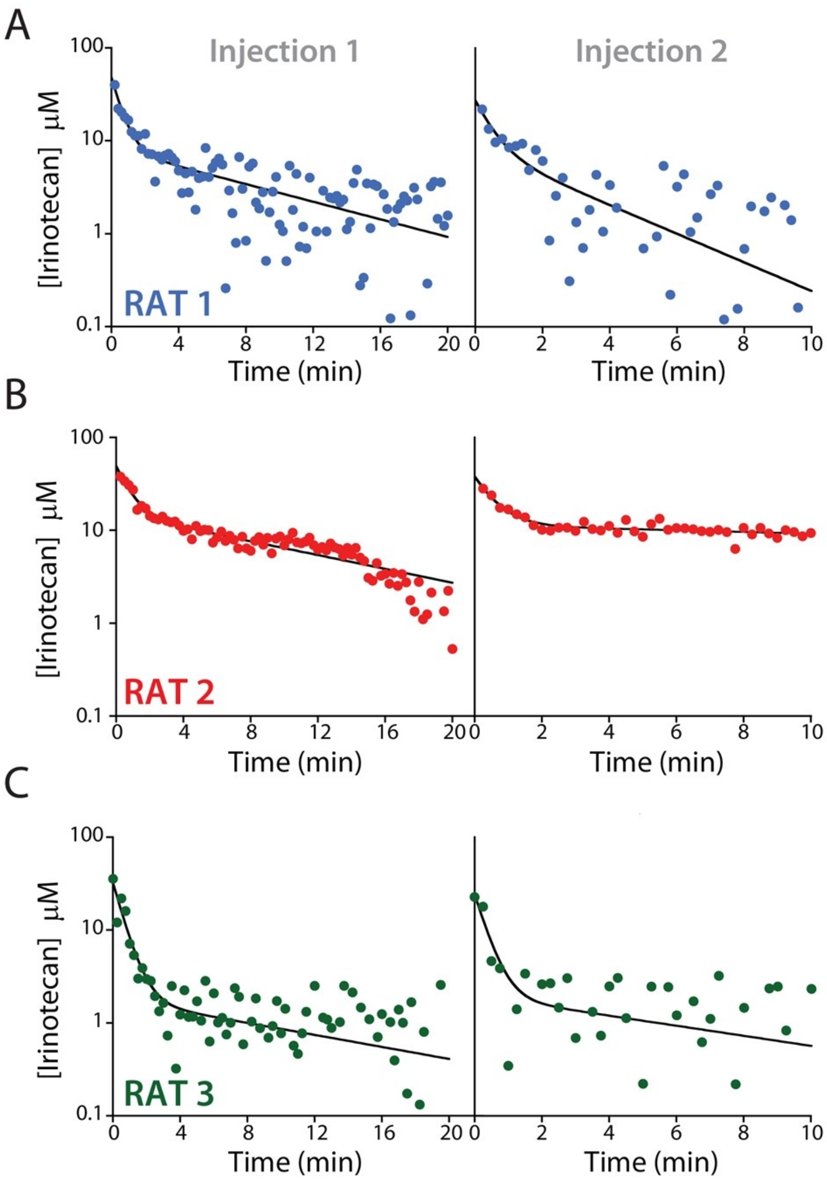

Figure S6. The precision of indwelling E-AB sensors is great enough to measure not only inter-animal pharmacokinetic variability but also variability within a single individual over time. The pharmacokinetic profiles shown are for sequential injections (20 mg/kg and 10 mg/kg separated by ~25 min) of irinotecan in three different rats (A, B, and C). The bold black lines represent the fit of each injection dataset to a two-compartment pharmacokinetic model (see materials and methods). See table S2 for the relevant pharmacokinetic parameters.

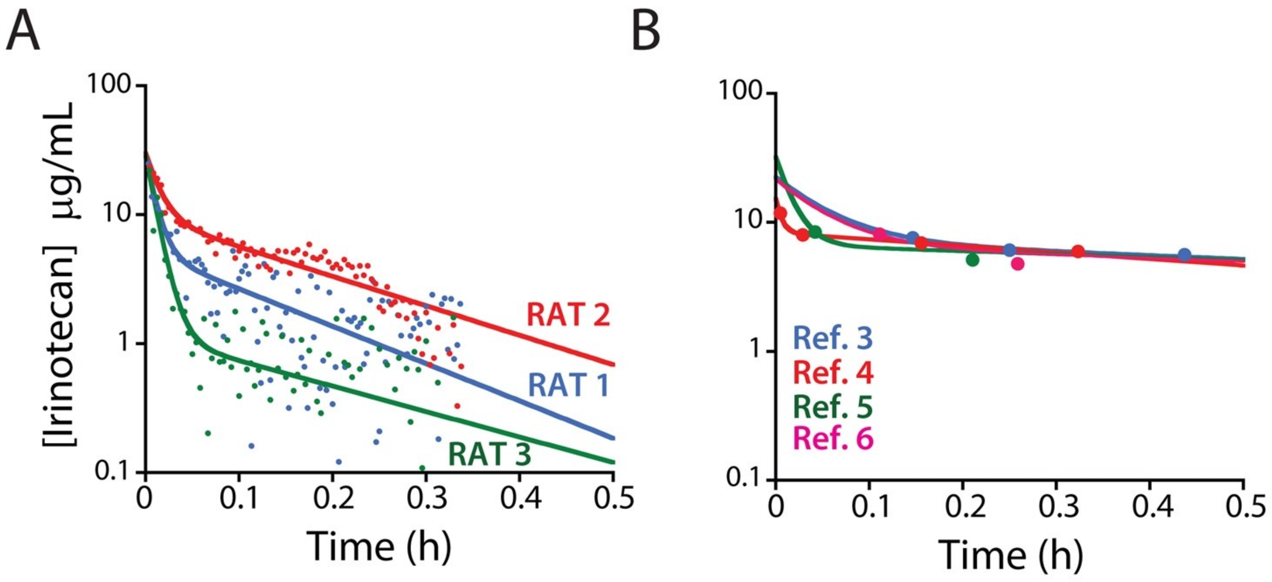

Figure S7. A comparison of (A) our real-time, high frequency measurements with (B) previously published studies of inrinotecan pharmacokinetics. For this we identified studies performed using the same intravenous injection (20 mg/kg) and the same animal species,[10-13] extracting the data from the referenced material using WebPlotDigitizer (http://arohatgi.info/WebPlotDigitizer/). Plotting the extracted data on the same unit scales as our collected data clearly indicates the difference between the estimated pharmacokinetic parameters.

References:

1. H. Fujita, Y. Imaizumi, Y. Kasahara, S. Kitadume, H. Ozaki, M. Kuwahara and N. Sugimoto, *Pharmaceutical*s, 2013, **6**, 1082-1093.
2. A. Idili, K. W. Plaxco, A. Vallée-Bélisle and F. Ricci, *ACS Nano*, 2013, **7**, 10863-10869.
3. Y. Xiao, R. Y. Lai and K. W. Plaxco, *Nat. Protoc.*, 2007, **2**, 2875-2880.
4. N. Arroyo-Currás, J. Somerson, P. A. Vieira, K. L. Ploense, T. E. Kippin and K. W. Plaxco, *Proc. Natl. Acad. Sci. USA,* 2017, **114**, 645–650.
5. N. Arroyo-Curras, K. Scida, K. L. Ploense, T. E. Kippin and K. W. Plaxco, *Anal. Chem.*, 2017, **89**, 12185−12191.
6. Committee for the Update of the Guide for the Care and Use of Laboratory Animals. (2011) Guide for the care and use of laboratory animals, 8th ed., The National Academies Press.
7. R. Urso, P. Blardi and G. Giorgi, *Eur. Rev. Med. Pharmacol. Sci.,* 2002, **6**, 33-44.
8. J. N. Zadeh, C. D. Steenberg, J. S. Bois, B. R. Wolfe, M. B. Pierce**,** A. R. Khan, R. M. Dirks and N. A. Pierce, *J. Comput. Chem.,* 2011, **32**, 170–173.
9. R. J. White and K. W. Plaxco, *Anal. Chem.,* 2010, **82**, 73-76.
10. N. Kaneda and T. Yokokura, *Cancer Res.,* 1990, **50**, 1721-1725.
11. N. Kaneda, H. Nagata, T. Furuta and T. Yokokura, *Cancer Res.,* 1990, **50**, 1715-1720.
12. T. Bansal, A. Awasthi, M. Jaggi, R. K. Khar and S. Talegaonkar, *Talanta,* 2008, **76**, 1015-1021.
13. T. Bansal, G. Mishra, M. Jaggi, R. K. Khar and S. Talegaonkar, *Eur. J. Pharm. Sci.,* 2009, **36**, 580-590.
